# *Chlamydia* Species-Dependent Differences in the Growth Requirement for Lysosomes

**DOI:** 10.1371/journal.pone.0016783

**Published:** 2011-03-08

**Authors:** Scot P. Ouellette, Frank C. Dorsey, Simon Moshiach, John L. Cleveland, Rey A. Carabeo

**Affiliations:** 1 Centre for Molecular Microbiology and Infection, Division of Cell and Molecular Biology, Imperial College, London, United Kingdom; 2 Department of Cancer Biology, The Scripps Research Institute, Scripps Florida, Jupiter, Florida, United States of America; 3 Department of Tumor Cell Biology and Genetics, St. Jude Children's Research Hospital, Memphis, Tennessee, United States or America; University of California, San Francisco, University of California, Berkeley, and the Children's Hospital Oakland Research Institute, United States of America

## Abstract

Genome reduction is a hallmark of obligate intracellular pathogens such as *Chlamydia,* where adaptation to intracellular growth has resulted in the elimination of genes encoding biosynthetic enzymes. Accordingly, chlamydiae rely heavily on the host cell for nutrients yet their specific source is unclear. Interestingly, chlamydiae grow within a pathogen-defined vacuole that is in close apposition to lysosomes. Metabolically-labeled uninfected host cell proteins were provided as an exogenous nutrient source to chlamydiae-infected cells, and uptake and subsequent labeling of chlamydiae suggested lysosomal degradation as a source of amino acids for the pathogen. Indeed, Bafilomycin A1 (BafA1), an inhibitor of the vacuolar H^+^/ATPase that blocks lysosomal acidification and functions, impairs the growth of *C. trachomatis* and *C. pneumoniae*, and these effects are especially profound in *C. pneumoniae*. BafA1 induced the marked accumulation of material within the lysosomal lumen, which was due to the inhibition of proteolytic activities, and this response inhibits chlamydiae rather than changes in lysosomal acidification *per se*, as cathepsin inhibitors also inhibit the growth of chlamydiae. Finally, the addition of cycloheximide, an inhibitor of eukaryotic protein synthesis, compromises the ability of lysosomal inhibitors to block chlamydial growth, suggesting chlamydiae directly access free amino acids in the host cytosol as a preferred source of these nutrients. Thus, chlamydiae co-opt the functions of lysosomes to acquire essential amino acids.

## Introduction

Intracellular bacterial pathogens must overcome a battery of host cell mechanisms that block bacterial growth. One such hurdle is phagolysosomal degradation, wherein the host cell directs internalized bacteria into vesicle that is delivered to the acidic lysosome for degradation. Accordingly, intracellular pathogens have evolved mechanisms to avoid this process, including diversion of the vesicle away from the endocytic pathway (*e.g.*, *Chlamydia* [1] and *Legionella* [2]), lysing the vesicle and growing in the cytosol (*e.g.*, *Rickettsia* [3], *Shigella* [4], and *Listeria* [5]), delaying or blocking the maturation of the phagosome (*e.g.*, *Mycobacterium* [6], and *Salmonella* [7]) or using the acidified environment as a cue for growth and development (*e.g.*, *Coxiella* [8], and *Brucella* [9]). Collectively, these data have led to the prevailing dogma that intracellular pathogens avoid, disrupt, or subvert the lysosome. However, there is a dearth of information regarding how organisms such as chlamydiae, which reside in a membrane-bound vacuole, acquire essential nutrients and how they interact with the lysosome.

Chlamydiae are obligate intracellular bacterial pathogens that cause a broad spectrum of diseases in humans and animals. For example, *Chlamydia trachomatis* (Ctr) is the leading cause of bacterial sexually transmitted diseases in developed countries [10] and of preventable blindness (trachoma) in developing nations [11]. Further, *C. pneumoniae* (Cpn) causes community-acquired pneumonia and other respiratory tract diseases [12]. More importantly, these pathogens cause chronic sequelae when not diagnosed or left untreated, where Ctr infections lead to pelvic inflammatory disease, tubal factor infertility [13], and reactive arthritis [14], and where Cpn infections may lead to atherosclerosis [15], adult-onset asthma [16], and other chronic conditions [17].

These highly successful pathogens utilize a biphasic life cycle that alternates between an infectious extracellular, but metabolically inert, elementary body (EB) and a non-infectious intracellular, but metabolically active, reticulate body (RB) (for review see [18]). Following attachment to a susceptible host cell the EB is internalized by pathogen-directed processes into a vesicle that is then rapidly modified to avoid lysosomal fusion. Specifically, shortly after uptake the EB differentiates to the RB, which then grows and divides within the diverted vesicle. This modified vacuole, termed an inclusion, contains chlamydial-derived proteins in its membrane. Chlamydiae subvert various host cell processes that allow the bacteria to survive in the cell and to expand the inclusion as RBs multiply. Finally, in an asynchronous manner, RBs differentiate back to EBs, which are then released from the host cell.

Host cell interactions with the inclusion have been frustratingly difficult to identify. The chlamydial inclusion is a neutral pH compartment freely permeable to ions [19] but impermeable to compounds as small as 520 Da [20]. Previous studies have suggested that chlamydiae do not depend on endosomal acidification for growth, as no effects on inclusion formation were detected in the presence of acidification inhibitors [1], [21]. Further, markers of the endocytic pathway (*e.g.*, EEA-1, Rab5, Rab7, and LAMP1/2; [22]) are not associated with the inclusion. Rather, *Chlamydia* appears to insert itself into exocytic and recycling pathways as it is can intercept sphingomyelin- [23] and cholesterol- [24] containing vesicles and since the inclusion co-localizes with markers of recycling endosomes [22]. However, nascent host cell-derived proteins have not been detected in the inclusion membrane, suggesting that *Chlamydia* exploits as yet undefined trafficking pathways [25]. This is supported by findings that disrupting exocytic pathways does not inhibit chlamydial growth; thus, other pathways are used to obtain nutrients.

It has been assumed that chlamydiae acquire nutrients from the host cell by directly transporting free amino acids, nucleotides, and other small molecules, or that they acquire these and possibly other nutrient sources from exocytic vesicles. Diverting the free amino acid pool to the chlamydiae by inhibiting *de novo* host protein synthesis enhances pathogen growth, suggesting chlamydiae preferentially rely on the transport of free amino acids into the inclusion rather than intercepting vesicles to acquire these nutrients [26], [27]. We reasoned, by virtue of its degradative functions, that the lysosome serves an obligate role for chlamydiae - that of a source of amino acids. Here, we report that *Chlamydia* indeed require lysosomal proteases and lysosomal-mediated degradation of exogenous proteins for intracellular growth, even under growth conditions in which free amino acids were present in the extracellular medium, ready for transport. Thus, the growth inhibitory effects observed in chlamydiae were not due to the failure to replenish the cytosolic pool of amino acids. Rather, it is likely due to the depletion of amino acids and/or oligopeptides within the lysosomal compartments. Additionally, we show a marked difference between chlamydial species and strains and that their requirement for lysosomal activity depends on their growth rate, where the slowest growing species and strains are the most dependent on the lysosome. These findings support a model whereby the degradation of cargo derived from the endocytic pathway by the lysosome provides essential nutrients for growth of *Chlamydia*.

## Results

### The chlamydial inclusion is in close apposition to lysosomes throughout the developmental cycle

**Figure 1:**
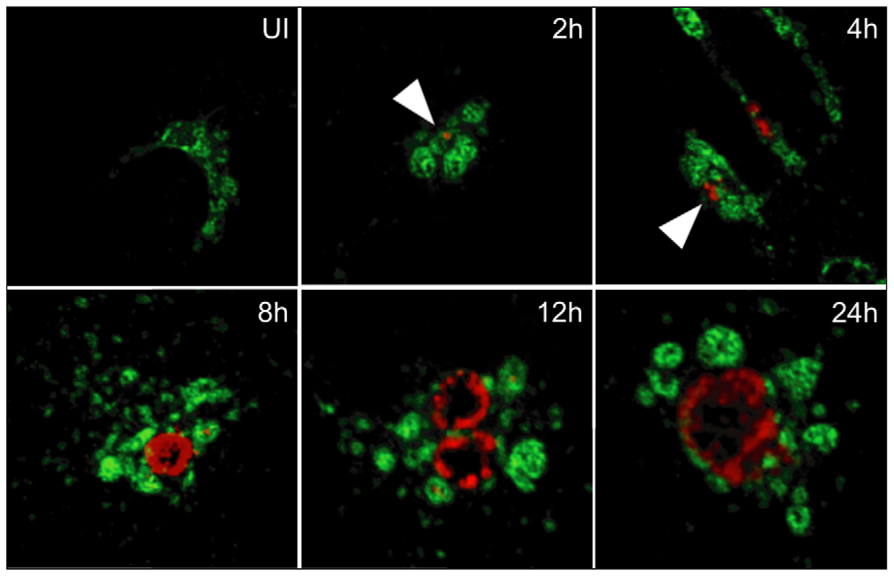
The chlamydial inclusion is in close apposition to lysosomes throughout its developmental cycle. Cells were transfected with LAMP1-YFP (green) and were infected with *Chlamydia trachomatis* serovar L2 (red) or left uninfected (UI). At indicated times post-infection, cells were fixed and stained for chlamydiae. Arrowhead indicates an early chlamydial inclusion.

Chlamydial inclusions localize to the microtubule organizing center (MTOC; [28]). Interestingly, lysosomes also localize to the perinuclear region of the cell, and thus we investigated the relative subcellular localization of chlamydial inclusions and their association with lysosomes. To monitor lysosomal morphology and localization, HEp-2 cells were transfected with a vector driving the expression of LAMP1-YFP, a lysosomal transmembrane protein fused in-frame with yellow fluorescence protein (YFP), and these cells were then infected with Ctr and assessed for their localization by confocal microscopy. Strikingly, chlamydial inclusions were always found in close proximity to lysosomes throughout their developmental cycle (Fig. 1), although the LAMP1-YFP marker never co-localized to the inclusion membrane [1].

### Lysosomal degradation products are transferred to chlamydiae

**Figure 2:**
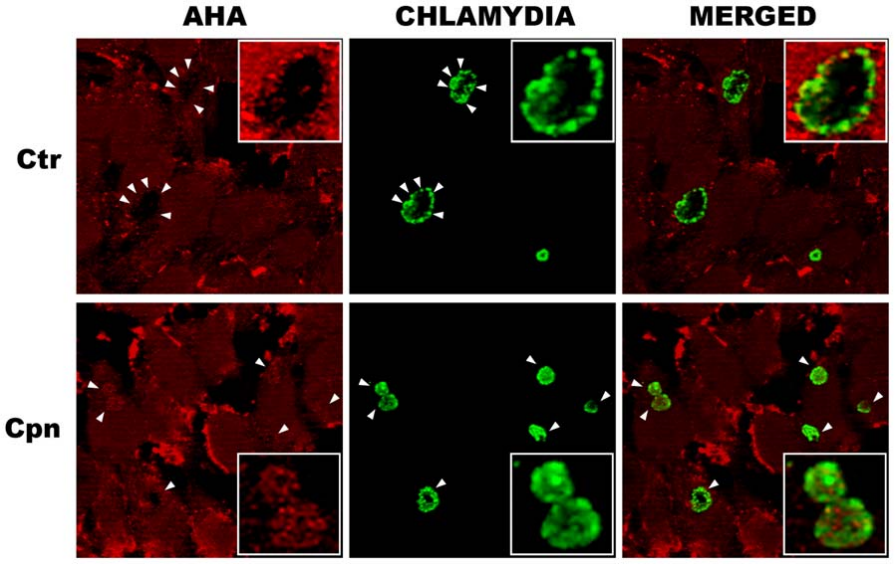
Lysosomal degradation products are transferred to chlamydiae. AHA-labeled protein was fed to infected cells, and cells were subsequently fixed and processed for confocal microscopy. Chlamydiae were counter-stained with an antibody to facilitate identification of AHA label within chlamydiae. For Ctr, arrowheads indicate individual organisms positive for labeling with AHA whereas for Cpn, arrowheads indicate inclusions with multiple organisms. Note the intensity of the stain in the zoomed insets and the more uniform staining of Cpn organisms compared to Ctr. Bright staining on the cell surface is AHA-labeled protein lysate that was not endocytosed.

Given the close apposition of chlamydial inclusions to lysosomes during the developmental cycle, we hypothesized that lysosomal proteolytic activity, which catabolizes polypeptides to oligopeptides and free amino acids, may be a nutrient source for *Chlamydia*. It is well known that lysosomal and fluid phase markers are not transferred to the inclusion [1], [20], yet lysosomes do generate degradative products, such as oligopeptides for which chlamydiae have specific transport systems [29], [30]. To determine if such a transfer could occur, we assessed the ability of pre-labeled proteins to be incorporated into chlamydiae following lysosomal digestion. Click-iT® chemistry was employed, where newly synthesized proteins are metabolically labeled with the amino acid analogue azidohomoalanine (AHA) in place of methionine using AHA-supplemented methionine-free medium. AHA-containing proteins can be detected using a fluorescent alkyne-containing ligand, and the covalent reaction of fluorescent ligand to AHA-containing macromolecules occurs in the presence of copper catalysts [31]. Infected cells were fed with an AHA-labeled uninfected cell lysate, from which free AHA amino acids had been removed by filtration. Efficiency of removal of the free AHA amino acids by filtration was determined by spiking unlabeled lysates with free AHA and examining the amount of label associated with Cpn-infected cells. Under this condition, labeling was minimal (data not shown). The cells were fixed and processed for secondary labeling. Notably, AHA was easily visualized within chlamydial inclusions (Fig. 2). Cpn inclusions were uniformly labeled with AHA derived from pre-labeled protein whereas Ctr L2 inclusions were not consistently labeled. Not surprisingly, host cells were also labeled, as they endocytosed the protein lysate and metabolized its products, and the extracellular protein lysate was also visible. These data demonstrate for the first time that labeled amino acids and/or oligopeptides generated within the lysosome are transferred to chlamydiae. Furthermore, there is a species-dependent utilization of lysosomal pools of oligopeptides/amino acids, with *C. pneumoniae* acquiring a greater amount of label than *C. trachomatis*.

### Compromising lysosomal functions impairs chlamydial growth

The ability of *Chlamydia* to acquire lysosomal degradation products was surprising given previously published observations suggesting disruption of lysosomal activity has no effect on chlamydial growth [1], [21], [32]. Thus, we initiated studies to carefully assess whether lysosomal functions were required for chlamydial growth by three independent approaches that provide increasing specificity – a) inhibiting the vacuolar H^+^/ATPase by Bafilomycin A1 (BafA1) to disrupt the acidic environs and pH-dependent functions of the lysosomes; b) inhibiting the activity of a subset of lysosomal cathepsins using the CathInIII cathepsin inhibitor; c) depleting cells of TFEB, a regulator of cathepsin gene expression. All of these experiments were conducted in media supplemented with free amino acids to ensure that the cytosolic pool would not be depleted.

#### Bafilomycin A1

**Figure 3:**
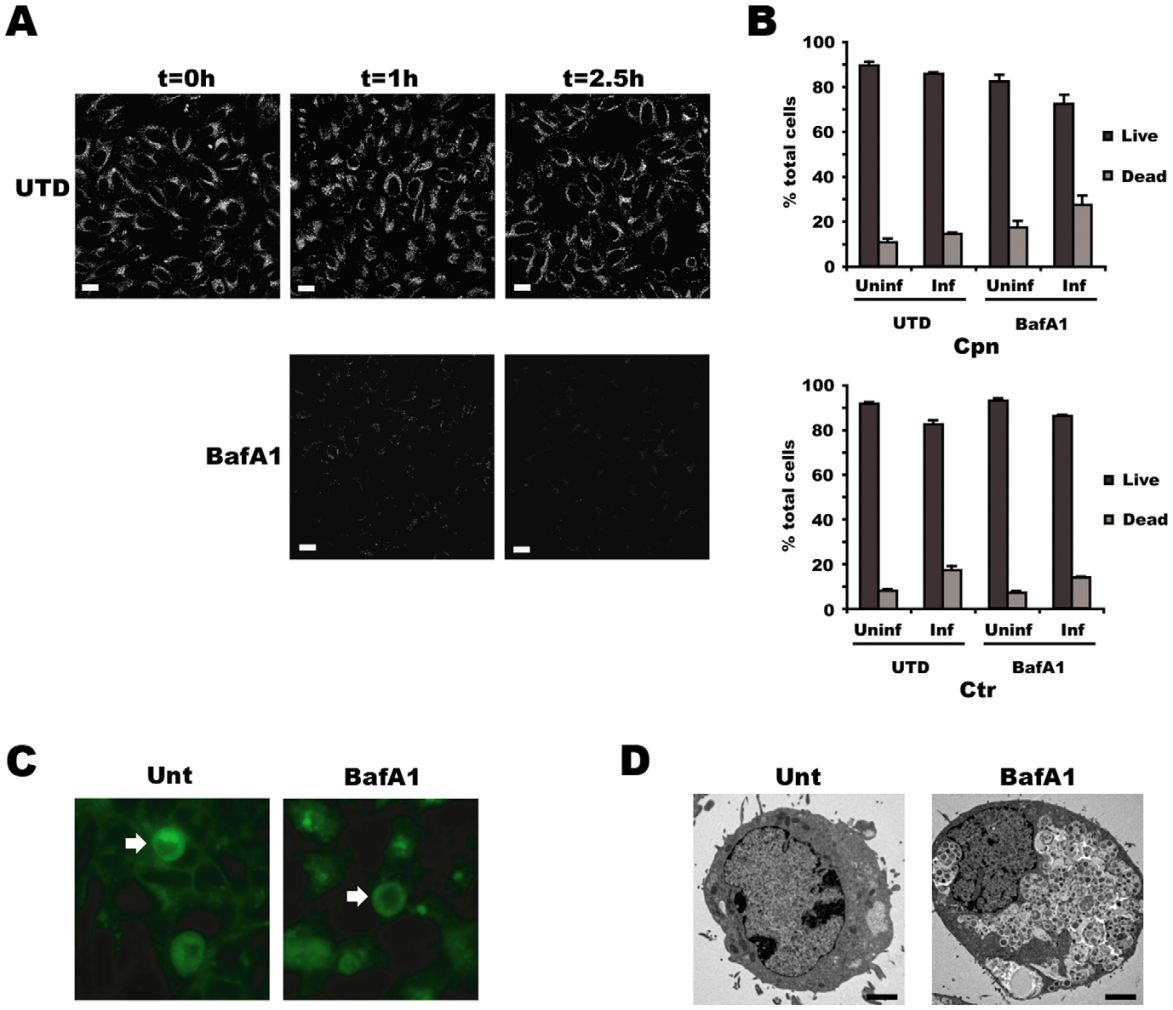
Bafilomycin A1 (BafA1) has selective effects on the host cell. (A) LysoTracker™ Red staining of untreated (UTD) or 100 nM BafA1-treated HEp-2 cells for the indicated times. Bar represents 10 µm. (B) Cell viability was measured by flow cytometry of Annexin/PI stained cells. Viability of uninfected and Cpn-infected cells +/− 100 nM BafA1 (top) and of uninfected and Ctr-infected cells +/− 100 nM BafA1 (bottom). (C) BafA1 does not disrupt uptake of fluorescent sphingomyelin by chlamydiae during BafA1 treatment. Cells were infected with Ctr L2 and pulsed with fluorescent ceramide, which is converted to sphingomyelin in the Golgi and subsequently transported by the exocytic pathway. Images were acquired at 630× magnification on an epifluorescent microscope. (D) Ultrastructural analysis of untreated and BafA1-treated uninfected HEp-2 cells. Note the presence of enlarged vesicular structures that contain undigested materials. Scale bar equals 1 µm.

The relatively broad specificity and potential cytotoxicity of BafA1 warranted a series of control experiments. Therefore, the increase in lumenal pH, effects on other trafficking events, namely sphingomyelin transport to the inclusion, and cytotoxicity to the host cells were carefully monitored, with the last by microscopy and fluorescence activated cell-sorting analysis of cell death by apoptosis (Annexin V) and loss of plasma membrane integrity (propidium iodide). Data from all of the control experiments, which involved treatment with 50 nM or 100 nM BafA1 and with a final DMSO concentration of 0.1%, indicated that BafA1 was indeed able to inhibit acidification (Fig. 3A), trafficking events known to be relevant to *Chlamydia* (i.e. transport of sphingomyelin from the trans-Golgi) appeared unaffected (Fig. 3C), and the levels of cytotoxicity observed were negligible (Fig. 3B). Additionally, treatment with BafA1 resulted in the accumulation of undegraded materials in the lysosomes (Fig. 3D).

**Figure 4:**
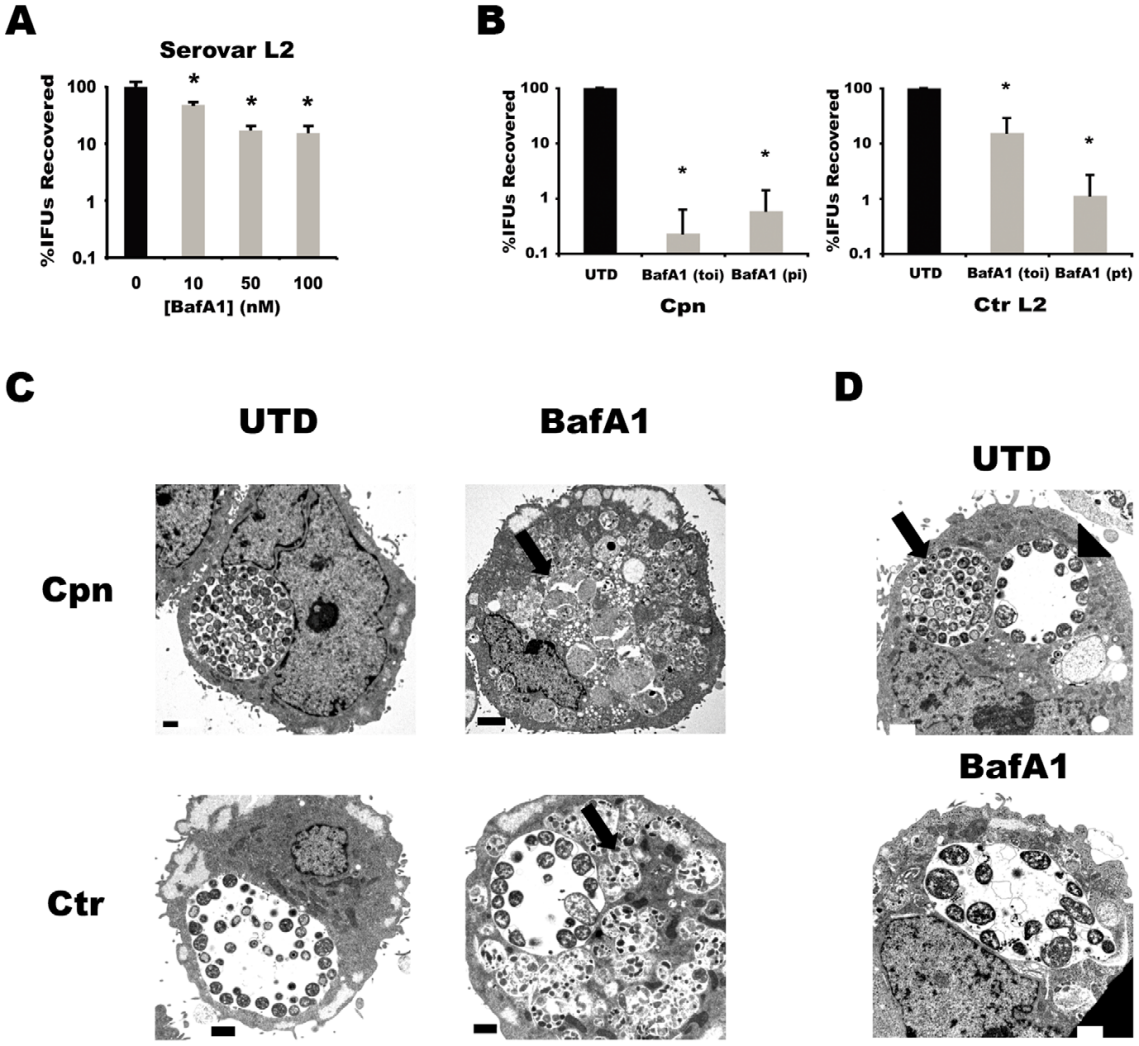
Impairing lysosomal functions blocks growth of *Chlamydia*. (A) IFU recovery of Ctr serovar L2 from cells treated with the indicated doses of BafA1. (B) 100 nM BafA1 was added to Cpn or Ctr at different times relative to infection: toi  =  time of infection, pi = 24 hr post-infection, pt = 24 hr pre-treatment. For IFU recovery experiments, data were pooled from at least three independent experiments with the average and standard deviation shown in percentage of the untreated control. Two-tailed Student's *t* tests assuming equal variance were performed for each experiment and *p* values combined using Fisher's Test for Combining *p* values. * indicates *p*<0.0001 for treatments compared to untreated control. (C) Ultrastructural analysis of untreated and BafA1 (100 nM)-treated Cpn- or Ctr-infected cells. Arrow indicates lysosome with undegraded material. Bars for cells infected with Cpn only (A and B) represent 2 µm whereas all others represent 1 µm. (D) BafA1 does not induce an anti-chlamydial effector. Cells were infected first with Cpn and then with Ctr to determine if Ctr could protect Cpn during BafA1 treatment, which would suggest a Ctr secreted factor in allowing growth. Arrow indicates Cpn inclusion whereas arrowhead indicates Ctr inclusion. The bottom panel shows a cell co-infected with Cpn- and Ctr L2 and treated with BafA1, with only a distinguishable Ctr inclusion present.

The effects of BafA1 treatment on the growth of *Chlamydia*, specifically *C. trachomatis* LGV serovar L2 were evaluated in a dose-response experiment, with BafA1 concentrations ranging from 0 to 100 nM (Fig. 4A). Ctr L2 was chosen because it was one of the least fastidious strains, and thus any effects observed with this serovar would likely be reproducible in more fastidious ones. It is clear that at the lowest dose of 10 nM, a statistically significant reduction in the yield of infectious elementary bodies could be observed, with higher levels of inhibition consistently observed with increasing BafA1 concentration. Thus, lysosomal acidification appears to be necessary for chlamydial growth. We observed a similar inhibition of chlamydial growth in cells treated with the lysosomotropic agent chloroquine (data not shown).

A direct comparison of relative susceptibility to BafA1 treatment between *C. trachomatis* and *C. pneumoniae* was conducted (Fig. 4B). Because of the different growth rates of the two chlamydial species, experiments that varied the treatment of cells with BafA1 relative to the time of infection were conducted to assess the temporal requirement by chlamydiae for lysosomal function, as well as to allow for a more detailed comparison of their relative susceptibilities. HEp-2 cells infected with *C. pneumoniae* or *C. trachomatis* were treated with BafA1 and IFU recovery was quantified. When 100 nM BafA1 was added at the time of infection, IFU recovery from Cpn-infected cells at 48 hr post-infection (p.i.) was reduced >99.5% or nearly 3-logs, whereas recovery from Ctr serovar L2-infected cells at 24 hr p.i. was reduced by approximately 90% (Fig. 4B).

Because the growth cycle of Cpn is 72–96 hours, we tested whether lysosomal functions were solely required for initial events following uptake of the EB or if this dependence was necessary throughout the developmental cycle. To this end, we delayed addition of BafA1 until 24 hr p.i. and then measured Cpn IFU recovery at 48 hr p.i. Under this scenario Cpn growth was still inhibited greater than 100-fold (Fig. 4B); thus, Cpn requires proper function of the endo/lysosomal system throughout its developmental program. Its greater sensitivity to BafA1 treatment relative to Ctr may reflect its greater reliance on the lysosome for nutrients.

Ctr completes its developmental cycle in approximately 48 hr, suggesting that the relative resistance of Ctr to BafA1 treatment could be accounted for in part by its more rapid growth. To test this notion, we pre-treated cells for at least one hour with BafA1 prior to infecting with Ctr, and then measured EB production at 24 hr p.i. Interestingly, Ctr growth was markedly diminished by pre-treatment with BafA1 (Fig. 4B). By varying the time of treatment, we discovered that proper lysosomal acidification is critical during the early life cycle of Ctr but is dispensable at later stages of development, whereas Cpn requires properly acidified lysosomes throughout its developmental cycle.

To investigate if inhibition of chlamydial growth by BafA1 may be related to acquisition of nutrients from the lysosome, as hinted by the labeling experiment (Fig. 4C), we performed an electron microscopic analysis of infected cells. We reasoned that nutrient deprivation should result in the establishment of aberrant reticulate bodies within intact inclusions [33]. Also, within the same cells, enlarged lysosomes filled with undigested material in BafA1-treated cells should be apparent. In untreated cells Cpn inclusions were readily detected with many RBs and some intermediate forms characteristically filling the inclusion space at 48 hr p.i. (Fig. 4C). Likewise, characteristic Ctr L2 inclusions were evident with bacteria juxtaposed to the inclusion membrane (Fig. 4C). Treatment with BafA1 led to a massive accumulation of undigested material within the lumen of lysosomes, which then swelled (Fig. 4B and D). Treatment with BafA1 completely abolished Cpn inclusion formation (Fig. 4C), demonstrating the requirement for a functional lysosome in the growth of this organism. In contrast, Ctr L2 inclusions were readily detected in BafA1-treated cells (Fig. 4C and 4D), yet here individual Ctr RBs were somewhat enlarged, suggesting impaired or delayed growth [33]. Finally, these effects correlated with the accumulation of undigested material in lysosomes, further suggesting that chlamydiae require the degradation of material within the lysosome for optimal growth. The failure to detect even abnormal inclusions suggests Cpn require lysosomal activity at early stages of infection.

That inclusions from Ctr, but not Cpn, were evident in BafA1-treated HEp-2 cells indicated that Ctr can partially overcome the effects of BafA1 and might produce a secreted factor that contributes to this resistance. To test this hypothesis, we assessed Cpn inclusion formation in co-infected cells treated with BafA1. Strikingly, after BafA1 treatment, only Ctr inclusions were apparent (Fig. 4D). Therefore, while Ctr is partially resistant to the effects of BafA1, this resistance does not allow Cpn growth in the presence of BafA1.

#### Cathepsin inhibitor

**Figure 5:**
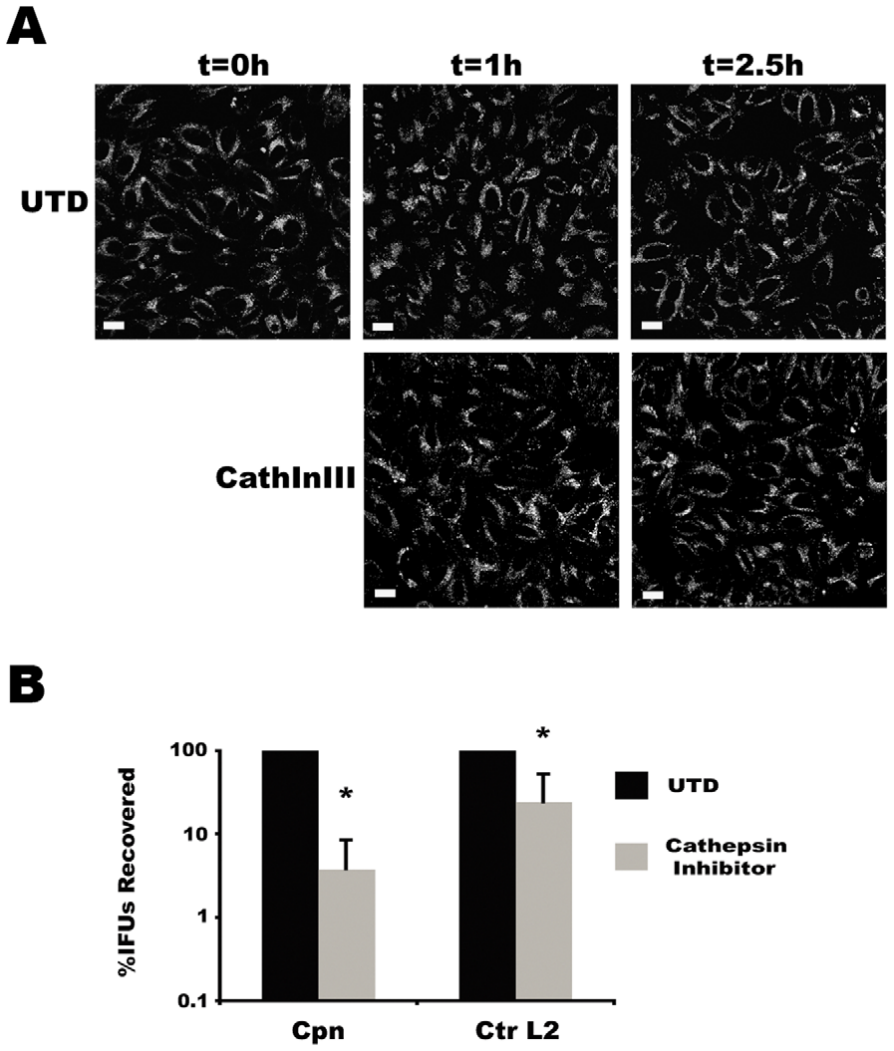
Cathepsin proteolytic activity is required for optimal growth of *Chlamydia*. (A) LysoTracker™ Red staining of untreated or 75 µM Cathepsin Inhibitor III-treated HEp-2 cells for the indicated times. Bar represents 10 µm. (B) Cathepsin Inhibitor III compromises chlamydial growth. This cathepsin inhibitor was titrated for its effect and was used at 40 µM. For IFU recovery experiments, data were pooled from at least three independent experiments with the average and standard deviation shown in percentage of the untreated control. Two-tailed Student's *t* tests assuming equal variance were performed for each experiment and *p* values combined using Fisher's Test for Combining *p* values. * indicates *p*<0.0001 for treatments compared to untreated control.

To refine further the results obtained from BafA1 treatment, we focused on a more specific aspect of lysosome function – proteolysis. Optimal activity of lysosomal proteases requires an acidic pH [34], and we hypothesized that the growth inhibitory effects of BafA1 on chlamydiae were simply due to the inhibition of lysosomal proteolysis. To test this, lysosomal proteolytic functions were compromised using an inhibitor of a subset of lysosome-associated cathepsins, Cathepsin Inhibitor III, which inhibits the lysosomal cysteine proteases cathepsins B, L, and S. Infected cells were treated with this cathepsin inhibitor and were then monitored for IFU recovery. This inhibitor did not affect lysosomal acidification (Fig. 5A). Thus, the growth inhibitory effects on chlamydiae of BafA1 could be linked directly with the inhibition of cathepsin activity, which is optimal at acidic pH. Notably, Cathepsin Inhibitor III blocked the growth of both Ctr L2 and Cpn, albeit somewhat less efficiently than BafA1 (Fig. 5B). Thus, the growth inhibitory effects on chlamydiae of BafA1 could be linked directly with the inhibition of cathepsin activity, which is optimal at acidic pH. The differences in the degree of chlamydial growth inhibition resulting from BafA1 and Cathepsin Inhibitor III treatments may reflect the continued activity of other lysosomal proteases that fall outside the target spectrum of the cathepsin inhibitor. There are ten cathepsins encoded in the human genome [35], and thus, functional redundancies and/or compensatory activities likely explain the lower degree of inhibition by Cathepsin Inhibitor III, which targets cathepsins B, L, and S. Regardless, it can be concluded from the data that both Ctr and Cpn require lysosomal proteases.

#### TFEB knockdown

**Figure 6:**
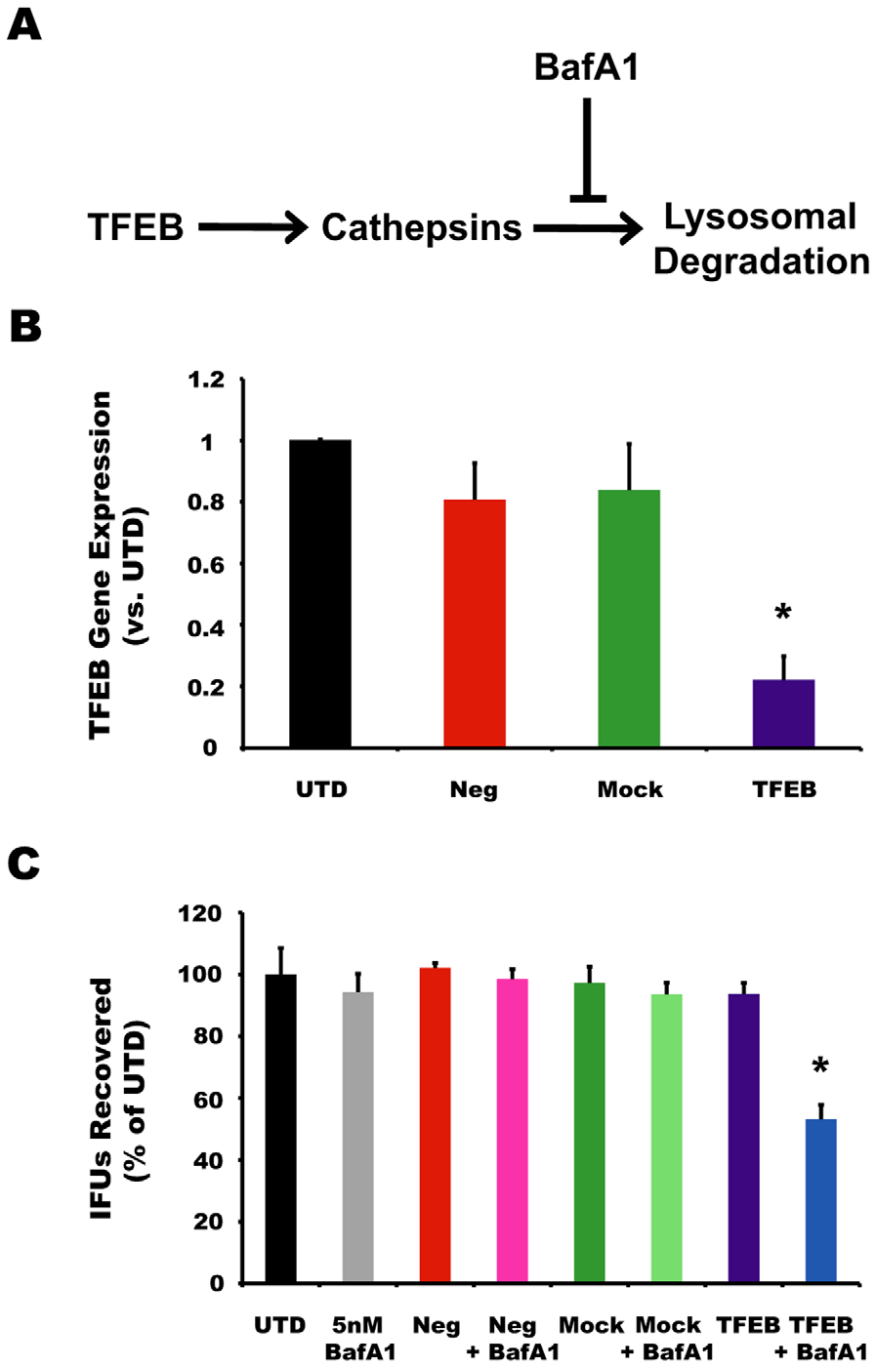
TFEB knockdown and sub-inhibitory doses of BafA1 block chlamydial growth. (A) Schematic of the role of TFEB in controlling lysosomal function. (B) Fold change in *TFEB* expression normalized to endogenous control *18S* rRNA expression in un-transfected (UTD), negative control siRNA transfected (Neg), mock-transfected (Mock), or TFEB siRNA transfected cells. Data represent 3 pooled experiments performed in triplicate. (C) Measurement of IFU recovery during transfection in the presence and absence of a sub-inhibitory dose (5 nM) of BafA1. Data represent at least 3 pooled experiments performed in triplicate. * indicates *p*<0.02 vs. all conditions.

A recent report identified TFEB as a master transcriptional regulator of lysosomal biogenesis and function [36]. Specifically, TFEB is necessary to induce the expression of cathepsins and many other genes required for the development and function of the lysosome (Fig. 6A). By contrast, BafA1 acts to disrupt the intralumenal environment of the lysosome to inhibit its function. Therefore, we tested whether depletion of TFEB by siRNA would impair chlamydial growth. Greater than 75% selective knockdown of *TFEB* mRNA expression was achieved (Fig. 6B), and the levels of TFEB target *CtsD* encoding for cathepsin D were reduced by ∼50% (Fig. S1). Effects of TFEB knockdown were tested on Ctr serovar B, which grows more slowly and would thus be more likely to show an effect over the course of TFEB knockdown. Although there was a trend towards smaller and fewer inclusions in TFEB knockdown cells, the degrees of variation between independent trials fluctuated enough to affect statistic significance. TFEB knockdown had little effect on the yield of infectious particles (Fig. 6C). However, any hidden effects of TFEB knockdown were revealed by the addition of a sub-inhibitory dose (5 nM) of BafA1, which is 10- to 20-fold less than the doses used in the above experiments (Fig. 6C). It is possible that any effects on the lysosomes of a partial knockdown of TFEB expression compromised these organelles to a degree that rendered them more susceptible to a sub-inhibitory dose of BafA1.

Collectively, the different approaches used to affect lysosomal function with increasing specificities all inhibited the growth of *Chlamydia*, thus indicating a growth requirement by the pathogen for this degradative organelle.

### Autophagy, the cytosol, and the lysosome as potential sources of amino acids for chlamydia

To implicate the lysosome in nutrient acquisition by *Chlamydia*, we decided to manipulate three potential sources of amino acids in the cell – autophagy, the cytosol, and the lysosome. Shifting the balance from one source to the other may provide insights into utilization of these amino acid reservoirs.

#### The autophagy pathway

**Figure 7:**
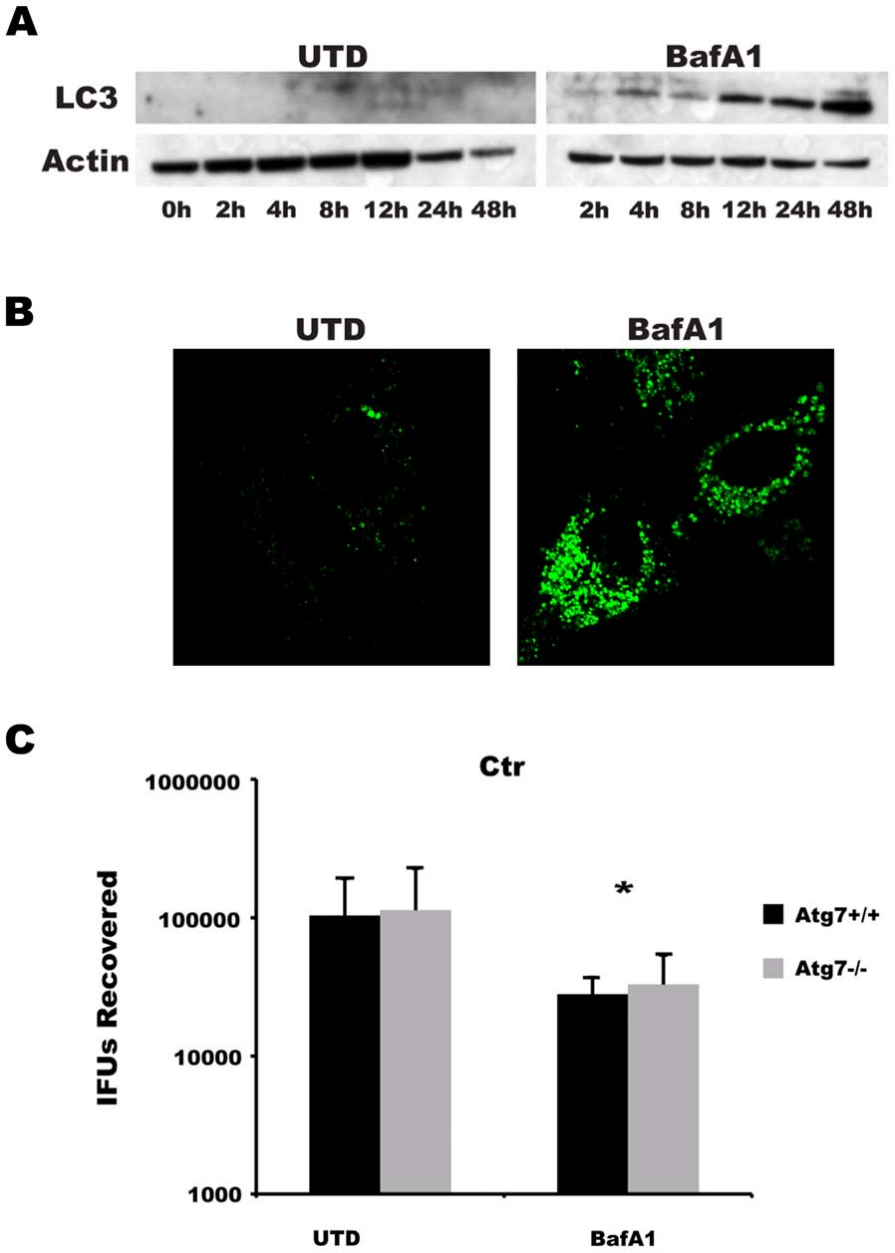
Autophagy is not a required source of nutrients for *Chlamydia*. (A) Western blot analyses demonstrated a marked accumulation of LC3 in BafA1-treated (100 nM) cells. Equal loading was confirmed by immunoblot with anti-actin antibody. (B) BafA1 treatment induced the accumulation of GFP-LC3 localized to vesicular structures in HEp-2 cells. (C) IFUs recovered from Ctr L2-infected, paired *Atg7* ^+/+^ or *Atg7* ^−/−^ MEFs that were left untreated (UTD) or were treated with BafA1 (50 nM). Data were pooled from three experiments performed in triplicate with the mean and standard deviation shown. Two-tailed Student's *t* tests assuming equal variance were performed for each experiment and *p* values were combined using Fisher's Test for Combining *p* values (* indicates *p*<0.005 for comparisons of treated and untreated cells). There was no significant difference in IFU recovery between *Atg7* ^+/+^ and *Atg7* ^−/−^ MEFs.

The lysosome is the nexus for degradation of both intracellular and extracellular proteins. Intracellular proteins can be delivered to lysosomes through a double-membrane vesicle called an autophagosome, whereas extracellular material is endocytosed and delivered to lysosomes through the endocytic pathway. Because of the intersection of these two pathways at the lysosome, nutrient pools derived from both would be susceptible to BafA1 treatment. Indeed, BafA1 effectively blocked both pathways, as cells treated with BafA1 had a marked accumulation of endocytosed BSA (data not shown) as well as LC3 (microtubule light chain 3), a protein that is both required for and degraded by the autophagy pathway [37] (Fig. 7A and 7B). The ability of BafA1 to block lysosomal-directed turnover of LC3 implies that it blocks other autophagy protein substrates, as least those of acid-activated proteases such as cathepsins. Since BafA1 abolished degradation of autophagic and endosomal cargo by the lysosome, we tested the role of autophagy in chlamydial growth, which has been suggested by others as necessary for growth of this pathogen [38]. To this end we evaluated the growth of Ctr in primary, early passage mouse embryonic fibroblasts (MEFs) lacking *Atg7*, an E1 enzyme that is essential for autophagosome formation [39], or paired wild-type MEFs. These studies were limited to Ctr, as Cpn does not grow in mouse cells [40]. Paired *Atg7* ^+/+^ and *Atg7* ^−/−^ MEFs were infected and either left untreated or were treated with 50 nM BafA1. Notably, Ctr L2 grew equally well in both wild type and *Atg7* ^−/−^ MEFs, and BafA1 treatment led to a 0.5 log difference in IFU for both lines compared to untreated cultures (Fig. 7C). Thus, Atg7 and autophagy are dispensable for chlamydial growth under conditions in which other sources of amino acids are intact. This observation is in agreement with others using *Atg5* knockout cells [41].

#### The cytosol

**Figure 8:**
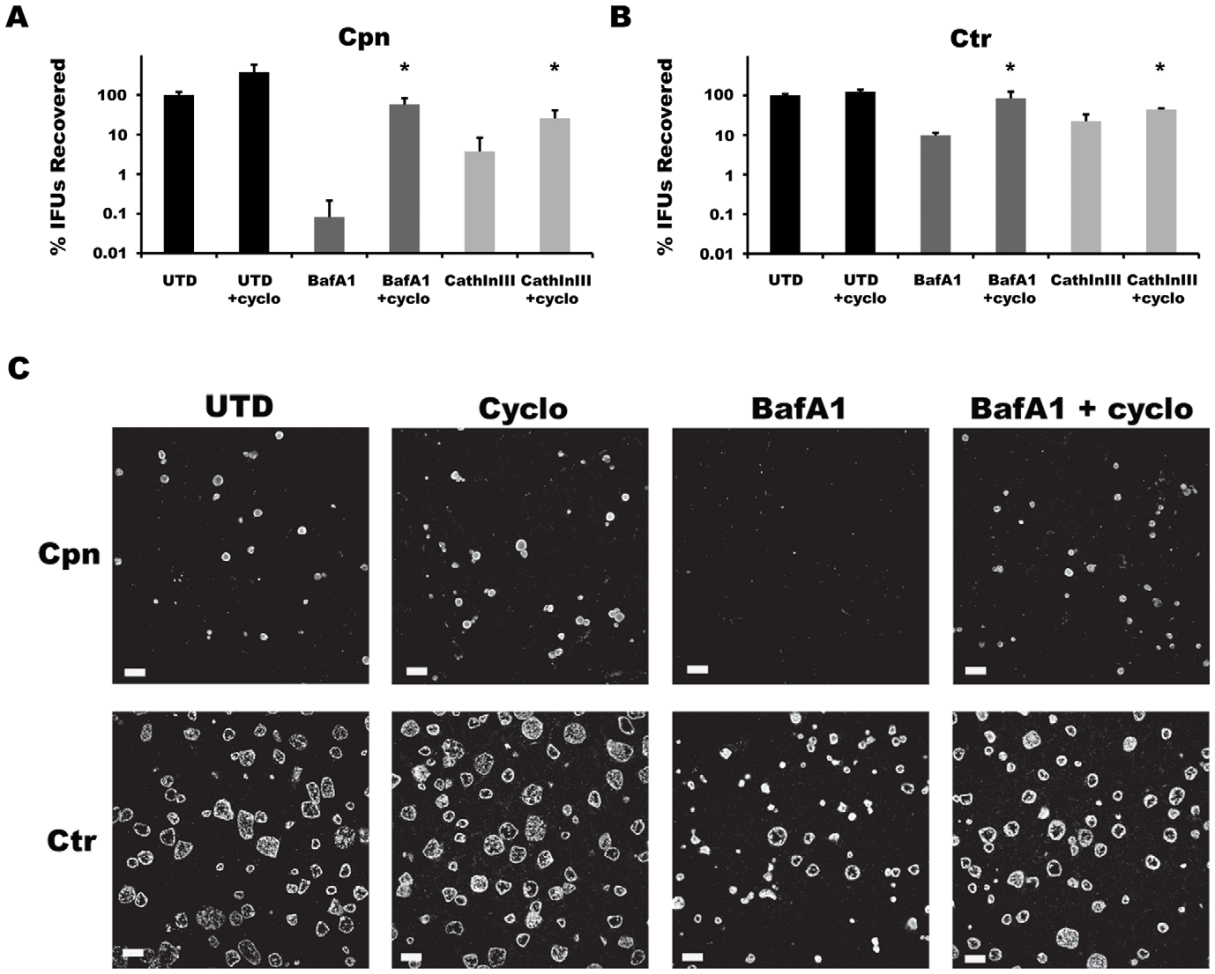
Cycloheximide rescues *Chlamydia* from the growth inhibitory effects of BafA1 and Cathepsin Inhibitors. IFUs recovered from (A) Cpn- or (B) Ctr L2-infected cells treated with BafA1 (100 nM) or Cathepsin Inhibitor III (40 µM) +/− cycloheximide (1 µg/ml) are shown. Data were pooled from at least three experiments performed in triplicate, with the mean and standard deviation shown as the percentage of the untreated control. Two-tailed Student's *t* tests assuming equal variance were performed for each experiment and *p* values combined using Fisher's Test for Combining *p* values (* indicates *p*<0.05 for cycloheximide treatments compared to relevant sample). (C) Immunofluorescence images of Cpn and Ctr inclusion in HEp-2 monolayers that were either untreated, BafA1-treated, or BafA1/cycloheximide-treated. Note the complete absence of mature Cpn inclusions in BafA1-treated cells.

The more fastidious chlamydial species and strains grow more slowly than others, and blocking eukaryotic protein synthesis with agents such as cycloheximide or emetine augments their growth [26], [27]. These observations were attributed to the elimination of competition from the host, with these agents effectively raising the pool of free amino acids that chlamydiae can access. We reasoned that increasing the cytosolic free amino acid pool, which is replenished by de novo biosynthesis and uptake from the extracellular environment via pinocytosis or import by specific transporters, would compensate for the loss of lysosome-associated oligopeptide/amino acid pools resulting from BafA1 or Cathepsin Inhibitor III treatment in infected cells. We therefore tested whether treatment of infected host cells with 1 µg/ml cycloheximide could override the inhibitory effects of BafA1 and Cathepsin Inhibitor III on the growth of Ctr and Cpn. Infected cells were collected and assessed for IFU recovery at 48 hr p.i. for Cpn and at 24 hr p.i. for Ctr L2. Cycloheximide did not restore lysosomal acidification in BafA1-treated cells as visualized by Lysotracker (data not shown). Remarkably, cycloheximide treatment rescued chlamydial growth in the presence of BafA1, and these effects were more profound for Cpn than for Ctr (Fig. 8A and 8B). A parallel immunofluorescence confocal images of the various experiment groups demonstrates the enhancing effects of cycloheximide on inclusion sizes of chlamydiae treated with BafA1 (Fig. 8C). The inhibitory effects of Cathepsin Inhibitor III on the growth of Cpn and Ctr were also reversed with cycloheximide. Thus, both chlamydial species have the ability to acquire free amino acids from the host, and for Cpn, this ability only becomes obvious when the host cell was prevented from utilizing the same pool. Taken together, the results indicate that chlamydiae have at least two sources of amino acids – those derived from within the lysosome and those that are transported from the extracellular environment into the host cytosol. Under conditions in which these two sources are intact, autophagy, likely the constitutive microautophagy, has minimal, if any role, in chlamydial growth. The ability of Cpn to grow and survive, in spite of its relatively poor ability to compete with the host cell for the cytosolic pool of amino acids compared to Ctr L2, is suggestive of its greater reliance on the lysosome.

### Exogenous delivery of BSA preferentially supports growth of *C. pneumoniae* in infected host cells deprived of amino acids

**Figure 9:**
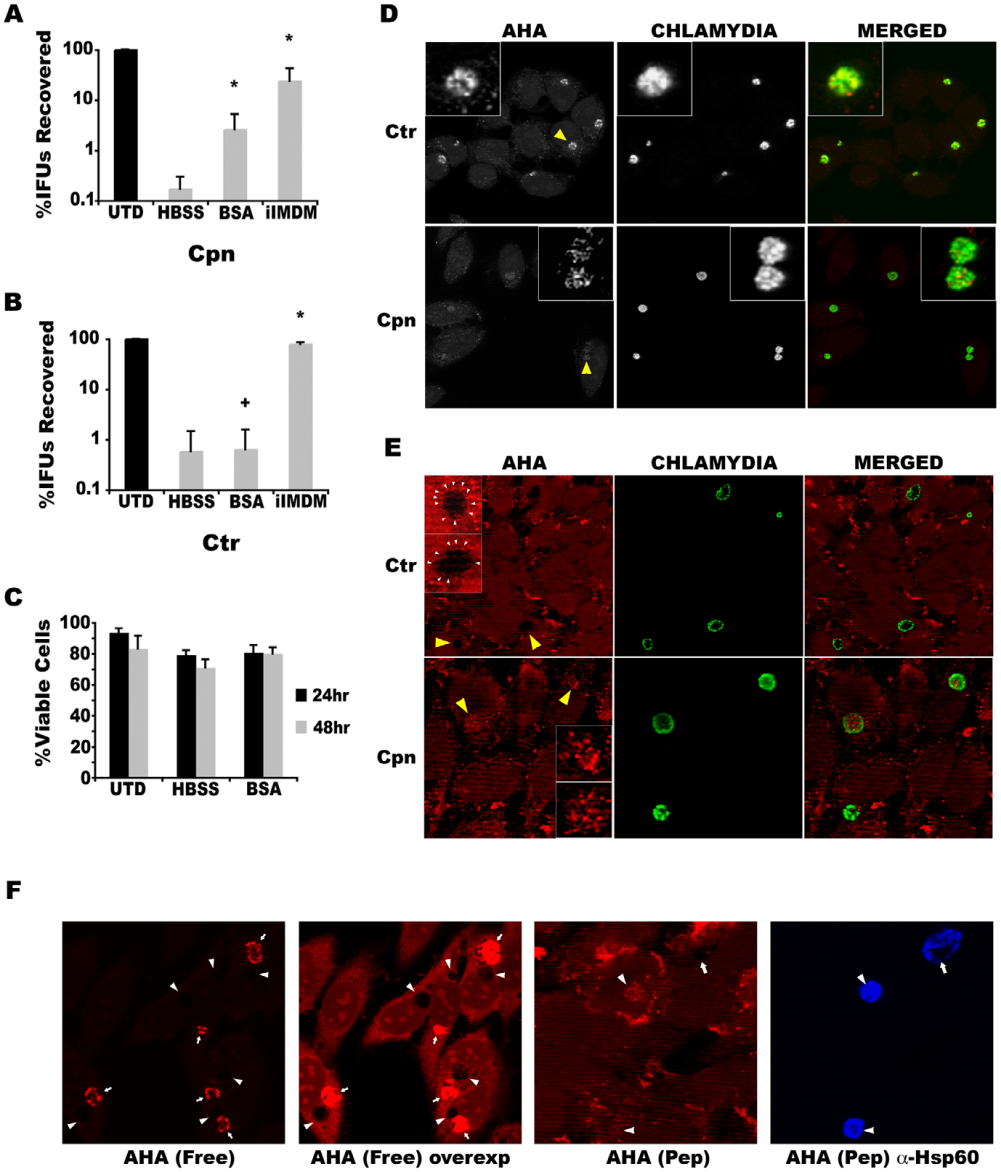
Lysosomally digested material promotes chlamydial growth in amino acid-free medium. (A) IFUs recovered from Cpn-infected cells grown in the indicated media are shown. (B) IFUs from Ctr L2-infected cells grown in the indicated media are shown. For (A) and (B), data were pooled from at least three experiments performed in triplicate with the mean and standard deviation shown as the percentage of the untreated control. Two-tailed Student's *t* tests assuming equal variance were performed for each experiment and *p* values combined using Fisher's Test for Combining *p* values (* indicates *p*<0.0001 for comparisons to HBSS alone; + indicates that two of three experiments testing the effects of exogenous BSA in Ctr infected cells were not significant). (C) Cell survival is not compromised by amino acid or serum deprivation. Viable cell numbers in the different growth media were determined at the indicated intervals by trypan blue exclusion. Data were pooled from two experiments with the mean and standard deviation shown. HBSS = Hank's Balanced Salt Solution. BSA = 5% BSA in HBSS. iIMDM = incomplete IMDM (lacking serum). (D–F) Amino acid preferences of Ctr and Cpn as revealed by labeling with AHA amino acid or AHA-labeled protein lysate fed to infected cells and imaged by confocal microscopy. (D) Pulse-labeling of chlamydiae with free AHA amino acid in the presence of cycloheximide. Note the weak labeling of Cpn compared to Ctr. (E) Infected cells were fed AHA-labeled protein cell lysate in the absence of cycloheximide. Note the comparative levels of stain between species. (F) Cells infected with both Cpn and Ctr were either pulse-labeled with free AHA amino acid or fed AHA-labeled protein cell lysate, as above, to directly compare the relative labeling efficiencies between the species. For panels (D–F), organisms were counterstained with antibody to facilitate visualization of organisms.

The data presented so far, which showed higher levels of AHA signal in Cpn inclusion within cells fed AHA-labeled polypeptides, sensitivity to BafA1 and Cathepsin Inhibitor III treatments, and enhancement of growth when the cytosolic pool of amino acids was liberated by cycloheximide treatment, suggested that Cpn is more dependent on lysosome-mediated degradation of proteins as a source of amino acids. To examine this greater dependency, we took advantage of the well-established observation that exogenous polypeptides, like horseradish peroxidase and bovine serum albumin are taken up and trafficked to the lysosome where they are degraded. When cultured in amino acid free medium, the endocytosis of exogenous proteins should support chlamydial growth. Thus, this experimental system is directly examining the role of the lysosome in providing amino acids/oligopeptides to chlamydiae in the absence of other sources and independent of pharmacological agents. To test this, infected cells were cultured in normal medium (with amino acids, glucose, and fetal calf serum), Hank's balanced salt solution (HBSS, no amino acids but normal levels of glucose), HBSS supplemented with 5% BSA (bovine serum albumin), or normal medium lacking serum (iIMDM). Importantly, the BSA used for these studies lacked free amino acids, as it was purified by alcohol precipitation, and there was no partial proteolysis as assessed by SDS-PAGE and Coomassie staining (data not shown). HEp-2 cells grown in all of these conditions were similarly competent for fluid phase uptake as demonstrated by dextran labeling (data not shown). As expected, Cpn and Ctr failed to grow and produce infectious particles in HEp-2 cells in the absence of amino acids. Only aberrant RB forms were detected in small inclusions (Fig. 9A and 9B, and data not shown). However, when supplemented with BSA, Cpn growth was partially restored as shown by the formation of infectious EBs. In contrast, BSA supplementation did not enhance Ctr L2 growth in HBSS. When growth was assessed in medium containing amino acids, but lacking exogenous peptides (*i.e.*, no serum), Ctr grew significantly better than Cpn (Fig. 9B). Since Cpn grows better in media supplemented with BSA than Ctr, we concluded that Cpn preferentially relies upon protein degradation for growth. This is quite remarkable given the sub-optimal ratio of amino acids that compose the BSA polypeptide compared to the amino acids present in the cell-culture optimized iIMDM.

Because depletion of amino acids and serum could provoke host cell death, we also monitored cell viability as above. The effects of amino acid depletion (cells cultured in HBSS +/− BSA) and the withdrawal of serum had modest effects on cell viability, which in all cases was greater than 70% by the completion of the experiments (Fig. 9C). Thus, the decrease in IFU recovery reflects diminished nutrient supplies for the chlamydiae as opposed to cell death. Because BSA could only be taken up by micro- and macropinocytosis, the restoration of Cpn growth could be explained parsimoniously by the transport to the lysosome of BSA for degradation and subsequent generation of amino acids and oligopeptides for chlamydial utilization.

The preferences of Cpn versus Ctr for sources of amino acids was also investigated by labeling with azidohomoalanine (AHA) as described above, either in the free form or incorporated into polypeptides prepared from cells incubated with this methionine homologue. Labeling with free AHA amino acid led to greater fluorescence signal appearing in Ctr inclusions versus Cpn inclusions (Fig. 9D). In contrast, labeling with AHA-labeled polypeptides yielded greater fluorescence within Cpn inclusions compared to Ctr inclusions (Fig. 9E).

This analysis was also applied to a Ctr/Cpn co-infection model, where we directly compared the ability of the two chlamydial species to obtain oligopeptides/amino acids in co-infected cells. HEp-2 cells were first infected with Cpn, followed by Ctr infection at 24 hr p.i. The infected monolayer was incubated with free AHA or AHA-labeled polypeptides prepared from labeled cell lysates and the samples were processed for Click-iT detection and subsequent confocal microscopy. Notably, within the same monolayer and in some cases within the same cell, Ctr inclusions were labeled more efficiently by free AHA than by AHA-labeled polypeptide (Fig. 9F). Furthermore, in direct comparison with Cpn inclusions (arrowheads, Fig. 5F), those of Ctr (arrows, Fig. 5F) had higher levels of label. This is in contrast to Cpn inclusions, which showed an opposite labeling profile and a clear preference for AHA-labeled polypeptides. Thus, Ctr and Cpn have inherent differences in the way their amino acid requirements are satisfied.

## Discussion

We have explored the potential role of the lysosome as a source of nutrients, specifically of oligopeptides and/or amino acids, in supporting the growth of chlamydiae. In this report, we demonstrated the transfer of azidohomoalanine (AHA), a methionine analog, to *Chlamydia* from metabolic labeling experiments with two different precursors – free AHA and AHA incorporated into polypeptides. Investigating the biological relevance of such transfer using inhibitors of lysosomal acidification and cathepsin proteases, and knockdown of a protein required for lysosome biogenesis, approaches with increasing specificities, revealed a role for this organelle in the growth of Ctr and Cpn, albeit to different extents. Importantly, we made a novel observation of the strain-dependent differences in co-opting amino acid sources – via endosome-lysosome degraded exogenous sources or pools of intracellular free amino acids.

Precisely how chlamydiae acquire nutrients from the infected host cell, particularly amino acids, has been unclear as there are, as established here, redundant pathways in operation. The host cell satisfies its amino acid requirements through de novo biosynthesis, transport of environmental amino acids via amino acid transporters, and through degradation of polypeptides. The host cell resorts to de novo synthesis using glucose as the precursor when environmental sources fall below a threshold. In other words, it is more energetically favorable to import pre-synthesized amino acids or peptides. Replenishment of amino acid pools via degradative pathways can be further classified with regard to the source of the polypeptides. Exogenous polypeptides are endocytosed and delivered to the lysosome where they are degraded by acid-activated proteases to oligopeptides and amino acids. On the other hand, endogenous polypeptides could be degraded by the proteasome or are delivered to the lysosome by autophagosomes.

We observed the close proximity of chlamydial inclusions and lysosomes. Based on this observation, we investigated the notion that *Chlamydia* obtains amino acids and/or oligopeptides from this organelle, which receives cargo from both endosomal and autophagosomal pathways. Autophagy was discounted as chlamydiae grew equally well in autophagy competent and deficient MEFs, and their growth was similarly impaired by BafA1 treatment of wild type and *Atg7* knockout cells.

The principal mode by which lysosomal functions contribute to chlamydial growth were revealed by both inhibitor and genetic studies. Firstly, BafA1 or cathepsin protease inhibitors impaired chlamydial growth, suggesting that the functions of acid-activated cathepsins provide the necessary oligopeptides and/or free amino acids for chlamydial growth. Secondly, knockdown of the host master lysosomal transcription factor TFEB, a known transcriptional activator of cathepsins [36], augmented the sensitivity of chlamydiae to and blocked their growth when treated with sub-inhibitory doses of BafA1. Collectively, these data implicate lysosomal cathepsins as necessary proteases for chlamydial growth. Ideally, this question would be addressed by RNA interference of the cathepsins, but the number of cathepsin genes in the human genome, of which there are ten [35], precludes such an approach. Finally, the requirement for lysosomal cathepsin activity is consistent with our studies showing transfer of exogenous pre-incorporated AHA-labeled polypeptides into chlamydial inclusions and that exogenous polypeptides could support the growth of chlamydiae. Thus, it is likely that polypeptides trafficked to the lysosome via the endosomal transport pathway are processed into small molecular weight oligopeptides and/or amino acids that are then transported out of the lysosome by specific transporters [42], [43], where they are sequestered by chlamydiae. It is important to note that infected cells were grown in the presence of extracellular amino acids, which could be transported by the host cell to maintain its cytosolic amino acid pool. Therefore, effects on growth inhibition of chlamydia by the different means of modulating the degradative function of the lysosome were likely due to the depletion of amino acids and/or oligopeptides within the lysosomal compartments, and not due to the failure of the lysosomes to replenish the cytosolic pool.

In what form are amino acids during uptake by *Chlamydia*? They could be free or in the form of soluble oligopeptides, both of which could be generated within the lumen of the lysosomes [42], [43]. Our data suggest Cpn utilizes the oligopeptide form as their growth was restored by media supplemented with BSA even though the amino acid composition of BSA is not ideal for chlamydial growth. This apparent preference for oligopeptides was highlighted by the observed relative inefficiency in acquiring free AHA amino acid. The Cpn genome contains 14 genes encoding for oligopeptide and dipeptide transporters [30]; hence this species has the capability of taking up oligopeptides from the host cytosol. Because di- and tri-peptides are less than 520 Da, they should freely diffuse through the inclusion membrane [20] allowing these transporters to import peptides from the inclusion lumen directly into the bacteria [45]; the proximity of inclusions to lysosomes increases the likelihood that these peptides will be sequestered by chlamydiae.

**Figure 10:**
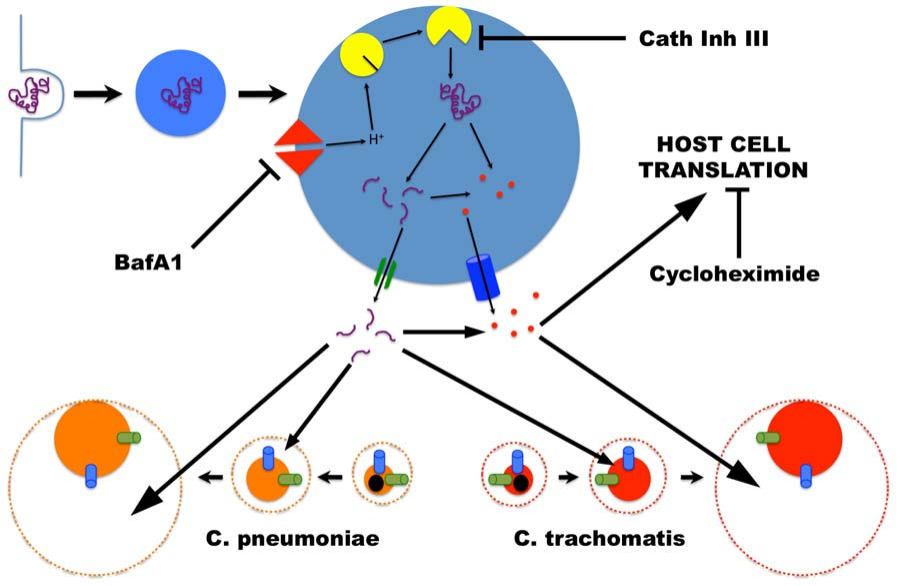
Chlamydial acquisition of amino acids. The data support a model where under normal growth conditions, chlamydiae compete with the host cell for free cytosolic pools of amino acids as well as those produced from lysosomal proteolytic degradation. EBs come pre-equipped with both amino acid and oligopeptide transporters. Early after infection and differentiation, the RB acquires amino acids likely using oligopeptide transporters. At later times, Ctr successfully competes with the host cell using its amino acid transporters whereas Cpn continues to rely on oligopeptide production from the lysosome. BafA1 abolishes lysosomal degradation of proteins. Cycloheximide blocks host cell usage of amino acids, thus increasing pools of free amino acids and ameliorating the effects of disrupting lysosomal-mediated protein degradation.

Cpn also possess a number of amino acid transporters [30], with their functionality demonstrated by the uptake of free AHA, albeit less efficiently than Ctr. Therefore, its inability to grow optimally in media supplemented with free amino acids may be due to factors other than transporter functionality – factors that may be related to growth rate and its ability to compete with the host. This is in contrast to the faster growing Ctr, which has a markedly different mode of amino acid utilization. Ctr was more dependent on the lysosome at the early stages of infection, with the establishment of larger and more mature inclusions correlating with resistance to BafA1 treatment. During maturation of the inclusion, we observed a nutrient switch from using oligopeptides originating from the lysosome to amino acids from the host cell cytosol, consequently conferring increasing independence from the lysosome, and thus increased BafA1 resistance. How this switch happens may be related to what is present within EBs and what is expressed during EB-to-RB differentiation. For example, DppA and DppD, components of a dipeptide transporter, are found in elementary bodies of Ctr [45]. We suspect that the reliance on the lysosome could be a way to bypass the competition from the host cell imposed on the chlamydiae. At early times in infection, an infecting EB, regardless of the species, has a small complement of ribosomes and would be at an immediate disadvantage to the host cell, and dependence on the lysosomes for oligopeptides and/or amino acids would overcome the competition at this stage of infection. This was certainly true for both Cpn and Ctr, which we found to be susceptible to BafA1 at the early stages of infection. With sufficient time, increased numbers of metabolically active chlamydial organisms would be poised to compete better with the host. Collectively, our findings support a model (Fig. 10) that explains how chlamydiae obtain amino acids from the host. Under normal conditions, fast-growing Ctr (with a division time of approximately two hours [46]) effectively compete with the host cell for amino acid pools and are thus less reliant on the lysosomal degradation pathway. By contrast, slower-growing Cpn (with a division time of approximately four hours [47]) are poor at competing with the host cell for free amino acids and thus they rely almost exclusively on the amino acids and peptide fragments generated through lysosomal proteolysis. Consequently, in the presence of BafA1, Cpn loses the primary source of amino acids for which it is adapted and thus cannot grow whereas Ctr can still utilize the free amino acid pools in the cytosol of the host.

Our data demonstrate the importance of lysosomal degradation in supporting chlamydial growth. We recognize that our studies challenge those of others who reported no effect of BafA1 on chlamydial growth [1], [21], [32]. The simplest explanation for this discrepancy is that we measured IFU recovery directly, which is a more accurate indicator of chlamydial growth and maturation, whereas others assessed inclusion development by microscopy, primarily for Ctr [1], [32]. With regards to the results of Al-Younes *et al.* [21], we suspect that the final concentration of the vehicle DMSO, rather than BafA1 affected the viability in their assays as neither we nor others [1], [32] have detected such detrimental effects of BafA1 on cell viability. We observed no overt toxicity of BafA1, which induces clear dose-dependent responses on chlamydial growth irrespective of host cell type, without appreciably affecting host cell survival. Indeed, HEp-2 cells exposed to both 100 nM BafA1 and cycloheximide were able to support near-normal growth of Chlamydia – a strong evidence that cell viability was not compromised by BafA1 treatment. Cytotoxicity was monitored carefully throughout the duration of the experiments by microscopy and by FACS analysis using two independent markers of cell death, annexin V (apoptosis) and propidium iodide (loss of plasma membrane integrity). The use of three criteria for cytotoxicity is well beyond what is typically found in scientific reports that have used BafA1.

In this report, we evaluated the roles of three potential sources of amino acids – authophagy, the cytosol, and the lysosome, and we fully acknowledge that there are other sources such as the proteasome, which we would have included in this investigation. Unfortunately, we were unable to directly test proteasomal involvement because blocking its activity for the duration of the chlamydial developmental cycle results in massive cell death. Rather, we stress that our data demonstrate that an alternative source of these nutrients for chlamydiae is the lysosome; with two distinct species demonstrating a contrasting utilization profile for this organelle.

Many intracellular bacterial pathogens appear to require acidification of the endosomal pathway for growth and survival within the host cell. For example, the growth of *Coxiella* and *Salmonella* is impaired by BafA1 [1], [8], [48], but, for each of these pathogens, the vacuole in which they reside is acidified, albeit to different extents. Acidification may serve as a nutrient source, as a cue for virulence expression, as an optimal environment for enzymes adapted to acidic environments, or possibly as some combination of all three. *Brucella* requires acidification soon after uptake by macrophages to activate virulence expression but does not require it thereafter [9]. Of these pathogens, only *Coxiella* is an obligate intracellular pathogen that has undergone extensive genome reduction leading to similar auxotrophies as *Chlamydia* [49]. Thus, it is reasonable that *Coxiella* would also require the proteolytic activity of cathepsins as an amino acid source. To our knowledge, this has not been tested. Indeed, this discovery for *Chlamydia* may stimulate more detailed studies on the role of the lysosome for other intracellular pathogens, especially those that are auxotrophic for essential amino acids. This report also has implications on the lysosome being a potential target for antibiotics given recent evidence that suggests targeting host cell pathways is an effective antibacterial strategy [50].

## Methods

### Organisms and cell culture

*Chlamydia pneumoniae* AR39 EBs were harvested from infected HEp-2 cell cultures at 35°C with 5% CO_2_, were purified by discontinuous density gradient centrifugation in Renografin (Bracco Diagnostics, Princeton, NJ), and were titered for infectivity by determining inclusion forming units (IFU). Similarly, EBs were harvested from *Chlamydia trachomatis* serovar L2 or B grown at 37°C with 5% CO_2_. HEp-2 cells were cultivated at 37°C with 5% CO_2_ in IMDM supplemented with 10% FBS, 2 mM L-glutamine, and 10 µg/mL gentamicin (all from Gibco/Invitrogen, Carlsbad, CA). Bafilomycin A1 and cycloheximide were purchased from Sigma Chemical Co. (St. Louis, MO) and Cathepsin Inhibitor III was purchased from EMD Biosciences (San Diego, CA). All other chemicals were from Sigma unless specified. HEp-2 cells stably expressing GFP-LC3 were made as described previously [53].

### Infection of cells with Cpn and Ctr

HEp-2 cells or MEFs were plated in 6-well culture plates at a density of 1×10^6^ cells per well. In a subset of wells, cells were plated onto glass coverslips for immunofluorescence microscopy. Approximately 18 hr later, confluent cell monolayers were rinsed with Hank's balanced salt solution (HBSS; Invitrogen), and, where indicated, 2 mL inoculum containing 2.6×10^6^ Cpn IFUs in SPG (0.25 M sucrose, 10 mM sodium phosphate, and 5 mM L-glutamic acid) was added to each well. Infected cell cultures were centrifuged in a Beckman Allegra 6R centrifuge for 15 min at room temperature, 400 x *g* then rocked for 15 min at 35°C without CO_2_. The inoculum was aspirated, and fresh supplemented IMDM with or without inhibitors was added to each well. Infected cultures were incubated at 35°C with 5% CO_2_. For Ctr 10^6^ IFUs (L2) or 2×10^6^ IFUs (B) were added directly to each well, and infected cells were incubated at 37°C with 5% CO_2_ unless co-infected with Cpn where they were cultured at 35°C. For all samples addition of inoculum to wells marks the time of infection (t = 0 hr). For BSA supplementation, cells were infected with Cpn as above, then incubated in HBSS, HBSS + 5% BSA, IMDM lacking serum (iIMDM), or normal medium. For Ctr, cells were pre-starved for 2 hr in HBSS prior to infecting in HBSS, 5% BSA, iIMDM, or normal medium.

### Transfection

5×10^4^ cells were plated on glass coverslips and allowed to attach overnight. Cells were transfected with 1 µg of LAMP1-YFP plasmid (a kind gift of Joel Swanson, University of Michigan) in Fugene 6 (Roche) according to the manufacturer's instructions in Optimem medium (Invitrogen). After 24 hr, cells were treated with or without 0.1 µM BafA1 for 24 hr. Cells were fixed in 4% paraformaldehyde in PBS overnight, rinsed, and viewed on an Olympus Fluoview 500 laser scanning confocal microscope. For siRNA experiments, cells were transfected with 9 pmol of 3 pooled Silencer siRNAs against TFEB (Applied Biosystems, Foster City, CA) or AllStars negative control siRNA (Qiagen) in HiPerFect Transfection Reagent (Qiagen) according to the manufacturer's instructions in Optimem medium. Cells were also mock-transfected (without siRNA) or left un-transfected as additional controls. After 20 hr transfection, cells were infected with 1 MOI of *C. trachomatis* serovar B in the presence or absence of 0.005 µM BafA1. At 30 hr post-infection, cells were harvested for IFU and qPCR analyses (see below).

### Quantification of IFUs from infected cell cultures

Medium was aspirated from the infected cells at the indicated times post-infection (p.i.), and 1 mL (6-well plate) or 250 µL (24-well plate) of SPG was added to each well. Cells were scraped and collected from each well into a 1.5 mL microfuge tube with 3 glass beads. Samples were vortexed for 45 sec and frozen at −80°C. Samples were titered for infectivity on fresh cell layers as described above to quantify the number of IFU per well.

### Immunofluorescence analyses

For LysoTracker™ staining, 0.5 µM LysoTracker™ Red (Invitrogen) was added directly to live cell cultures. Cells were incubated for 10 min then washed three times with prewarmed HBSS, at which point fresh media was added with or without inhibitors. Confocal fluorescent images were captured on an Olympus Fluoview 500 Laser Scanning Confocal Microscope.

### Flow cytometry

Cells infected with either Cpn or Ctr were trypsinized and collected at 48 hr p.i. in their culture medium, to preserve any dead cells that became detached from the culture dishes. Total cells were counted using a Vi-CELL™ (Beckman Coulter, Fullerton, CA)) cell counter system, and 10^6^ cells were stained using an Annexin V-FLUOS staining kit (Roche, Indianapolis, IN). Total numbers of live (no staining) and dead cells (Annexin V and/or PI staining) were quantified on a BD Biosciences LSR II flow cytometer.

### qPCR analysis

Total RNA from siRNA transfected, infected cells was collected as described by Ouellette *et al*. [46] using Trizol (Invitrogen). 1.5 µg RNA was reverse transcribed using Superscript III RT (Invitrogen) and random nonamer primers (New England Biolabs, Ipswich, MA) according to the manufacturer's instructions. Equal volumes of diluted cDNA were analyzed for *TFEB*, *Cathepsin D* (*CTSD*), and *18S* rRNA by quantitative PCR using pre-designed MGB probes (Applied Biosystems) on an ABI Prism 7300 Sequence Detection System (Applied Biosystems). The fold change in gene expression under each condition was determined by normalizing *TFEB* or *CTSD* expression to *18S* rRNA expression within each sample and comparing to un-transfected cells (ΔΔC_T_ method).

### Electron microscopy analyses

At 24 and 48 hr p.i., for Ctr and Cpn, respectively, cells from infected cultures were collected and centrifuged to a pellet for 5 min at 250 x *g*. For co-infection studies, cells were infected with Cpn as above, grown for 24 hr, then infected with Ctr and treated with or without BafA1. Cells were incubated for a further 24 hr prior to processing. Cell pellets were resuspended in 2% EM-grade glutaraldehyde diluted in PBS, transferred to a microfuge tube, and centrifuged to a pellet at 250 x *g* for 5 min. Cells were subsequently processed for EM as described elsewhere [52]. Briefly, cells were washed three times with PBS then fixed for 1 hr at room temperature in 1% osmium tetroxide diluted in PBS. Samples were dehydrated with a graded series of ethanol and embedded in Spurr's resin (Electron Microscopy Sciences, Ft. Washington, PA). 70 to 80 nm sections were cut, stained with uranyl acetate and lead citrate, and viewed on a Zeiss transmission electron microscope.

### Western blot analysis

At the times indicated, treated and untreated cells were collected and centrifuged, and protein was extracted from cells using RIPA buffer. Protein was quantified using a BCA protein assay (Pierce) according to the manufacturer's instructions. Equal amounts of protein were separated in a 10.5–14% gradient SDS-PAGE gel and transferred to nitrocellulose. Western blots were probed with both primary rabbit anti-LC3 [52] and mouse anti-actin (Sigma) antibodies. Appropriate secondary HRP-conjugated antibodies were used (Sigma). Blots were developed using Immobilon chemiluminescent substrate (Millipore Corp, Billerica, MA) and exposed to film.

### Isolation and culture of *Atg7* ^+/+^ and *Atg7* ^−/−^ MEFs

Embryos were harvested from timed matings of *Atg7* ^+/−^ mice at embryonic day E14.5 to generate littermate matched *Atg7^+/+^* and *Atg7* ^−/−^ embryos, and MEFs were isolated and genotyped for *Atg7* status as described [38].

### Metabolic labeling and uptake of AHA-labeled proteins

For metabolic labeling, infected cells were pre-starved for 1 hr in DMEM lacking cysteine (cys) and methionine (met). Chlamydiae were metabolically labeled for 8 hr with 50 µM azidohomoalanine (AHA; Invitrogen) in DMEM lacking cys and met (Invitrogen) and containing 10 µg/mL cycloheximide (Sigma) and fixed overnight with 4% PFA at 4°C. For uptake of AHA-labeled proteins, 2×175 cm^2^ flasks of uninfected cells were first metabolically labeled for 4 hr as above without cycloheximide. Cells were collected, centrifuged at 1500 rpm for 5 min at RT, and re-suspended in 1 mL PBS. Cells were freeze-thawed three times at −80°C and sonicated twice for 10 s to shear DNA and solubilize proteins. At 48 hr p.i. for Cpn or 12 hr p.i. for Ctr L2, the growth medium (IMDM) was removed from infected cells, and HBSS containing 10% AHA-labeled cell lysate (no cycloheximide) was added. After 9 hr of feeding, cells were fixed overnight with 4% PFA at 4°C. We tested whether filtration of the cell lysate with a 10 kDa cut-off filter was necessary to prevent residual AHA amino acid from labeling inclusions but found no effect. Similarly, we spiked unlabeled cell lysate with 12.5 µM AHA amino acid but found no labeling of chlamydiae under these conditions. The Click-iT Cell Reaction Kit with Alexa555-labeled alkyne (Invitrogen) was used according to the manufacturer's instructions for visualization of the AHA label. Chlamydiae were subsequently stained with primary mouse anti-chlamydial Hsp60 antibody and secondary goat anti-mouse Alexa647 antibody (Invitrogen).

### Image Processing

Images from experiments were imported into Adobe Photoshop CS2 and adjusted equally for contrast.

### Statistics

Two-tailed Student *t* tests assuming equal variance were performed within each experiment to determine *p* values (Microsoft Excel), which were combined for all experiments using Fisher's test for combining *p* values (MStat, University of Wisconsin, Madison, WI).

### Ethics Statement

This study was carried out in strict accordance with the recommendations in the Guide for the Care and Use of Laboratory Animals of the National Institutes of Health. The protocol was approved by the Institutional Animal Care and Use Committee of Scripps Florida (Permit Number: 09–025).

## Supporting Information

Figure S1 Expression of *TFEB* and *CTSD* over time during siRNA knockdown experiments. Cells were transfected as indicated or untransfected (UTD) and total RNA was collected. Expression of the genes was monitored by qPCR and normalized to 18*S* rRNA levels as described in Materials and Methods.
(TIF)

## References

[1] Heinzen RA, Scidmore MA, Rockey DD, Hackstadt T (1996). Differential interaction with endocytic and exocytic pathways distinguish parasitophorous vacuoles of *Coxiella burnetii* and *Chlamydia trachomatis*.. Infect Immun.

[2] Roy CR, Berger KH, Isberg RR (1998). *Legionella pneumophila* DotA protein is required for early phagosome trafficking decisions that occur within minutes of bacterial uptake.. Mol. Microbiol.

[3] Winkler HH, Miller ET (1982). Phospholipase A and interaction of *Rickettsia prowzekii* and mouse fibroblasts (L-929 cells).. Infect. Immun.

[4] High N, Mounier J, Prevost MC, Sansonetti PJ (1992). IpaB of *Shigella flexneri* causes entry into epithelial cells and escape from the phagocytic vacuole.. EMBO J.

[5] Beauregard KE, Lee KD, Collier RJ, Swanson JA (1997). pH-dependent perforation of macrophage phagosomes by listeriolysin O from *Listeria monocytogenes*.. J. Exp. Med.

[6] Sturgill-Koszycki S, Schlesinger PH, Chakraborty P, Haddix PL, Collins HL (1994). Lack of acidification in *Mycobacterium* phagosomes produced by exclusion of the vesicular proton-ATPase.. Science.

[7] Buchmeier NA, Heffron F (1991). Inhibition of macrophage phagosome-lysosome fusion by *Salmonella typhimurium*.. Infect. Immun.

[8] Hackstadt T, Williams JC (1981). Biochemical stratagem for obligate parasitism of eukaryotic cells by *Coxiella burnetii*.. Proc Natl Acad Sci U S A.

[9] Porte F, Liautard JP, Kohler S (1999). Early acidification of phagosomes containing *Brucella suis* is essential for intracellular survival in murine macrophages.. Infect. Immun.

[10] Centers for Disease Control and Prevention (2007). Sexually transmitted disease surveillance, 2006.

[11] Mabey DCW, Solomon AW, Foster A (2003). Trachoma.. Lancet.

[12] Grayston JT (1992). Infections caused by *Chlamydia pneumoniae* strain TWAR.. Clin. Infect. Dis.

[13] Brunham RC, MacLean IW, Binns B, Peeling RW (1985). *Chlamydia trachomatis*: its role in tubal infertility.. J. Infect. Dis.

[14] Taylor-Robinson D, Gilroy CB, Thomas BJ, Keat AC (1992). Detection of *Chlamydia trachomatis* DNA in joints of reactive arthritis patients by polymerase chain reaction.. Lancet.

[15] Saikku P, Leinonen M, Matilla K, Ekman MR, Nieminen MS (1988). Serologic evidence of an association of a novel *Chlamydia*, TWAR, with chronic coronary heart disease and acute myocardial infarction.. Lancet.

[16] Hahn DL (1995). Treatment of *Chlamydia pneumoniae* infection in adult asthma: a before-after trial.. J. Fam. Pract.

[17] Balin BJ, Gerard HC, Arking EJ, Appelt DM, Branigan PJ (1998). Identification and localization of *Chlamydia pneumoniae* in the Alzheimer's brain.. Med. Microbiol. Immunol.

[18] Abdel-Rahman YM, Belland RJ (2005). The chlamydial developmental cycle.. FEMS Microbiol. Rev.

[19] Grieshaber S, Swanson JA, Hackstadt T (2002). Determination of the physical environment within the *Chlamydia trachomatis* inclusion using ion-selective ratiometric probes.. Cellular Microbiology.

[20] Heinzen RA, Hackstadt T (1997). The *Chlamydia trachomatis* parasitophorous vacuolar membrane is not passively permeable to low-molecular-weight compounds.. Infect Immun.

[21] Al-Younes HM, Rudel T, Meyer TF (1999). Characterization and intracellular trafficking pattern of vacuoles containing *Chlamydia pneumoniae* in human epithelial cells.. Cell. Microbiol.

[22] Rzomp KA, Scholtes LD, Briggs BJ, Whittaker GR, Scidmore MA (2003). Rab GTPases are recruited to chlamydial inclusions in both a species-dependent and species-independent manner.. Infect Immun.

[23] Hackstadt T, Scidmore MA, Rockey DD (1995). Lipid metabolism in *Chlamydia trachomatis*-infected cells: directed trafficking of Golgi-derived sphingolipids to the chlamydial inclusion.. Proc Natl Acad Sci U S A.

[24] Carabeo RA, Mead DJ, Hackstadt T (2003). Golgi-dependent transport of cholesterol to the *Chlamydia trachomatis* inclusion.. Proc Natl Acad Sci U S A.

[25] Scidmore MA, Fischer ER, Hackstadt T (1996). Sphingolipids and glycoproteins are differentially trafficked to the *Chlamydia trachomatis* inclusion.. J Cell Biol.

[26] Hatch TP (1975). Competition between *Chlamydia psittaci* and L cells for host isoleucine pools: a limiting factor in chlamydial multiplication.. Infect. Immun.

[27] Ripa KT, Mardh PA (1977). Cultivation of *Chlamydia trachomatis* in cycloheximide-treated McCoy cells.. J. Clin. Microbiol.

[28] Grieshaber SS, Grieshaber NA, Hackstadt T (2003). *Chlamydia trachomatis* uses host cell dynein to traffic to the microtubule-organizing center in a p50 dynamitin-independent process.. J. Cell Sci.

[29] Stephens RS, Kalman S, Lammel C, Fan J, Marathe R (1998). Genome sequence of an obligate intracellular pathogen of humans: *Chlamydia trachomatis*.. Science.

[30] Read TD, Brunham RC, Shen C, Gill SR, Heidelberg JF (2000). Genome sequences of *Chlamydia trachomatis MoPn* and *Chlamydia pneumoniae AR39.*. Nucleic Acids Res.

[31] Dieterich DC, Lee JJ, Link AJ, Graumann J, Tirrell DA (2007). Labeling, detection and identification of newly synthesized proteomes with bioorthogonal non-canonical amino acid tagging.. Nat. Protoc.

[32] Schramm N, Bagnell CR, Wyrick PB (1996). Vesicles containing *Chlamydia trachomatis* serovar L2 remain above pH 6 within HEC-1B cells.. Infect. Immun.

[33] Beatty WL, Morrison RP, Byrne GI (1994). Persistent chlamydiae: from cell culture to a paradigm for chlamydial pathogenesis.. Microbiol Rev.

[34] Ishidoh K, Kominami E (2002). Processing and activation of lysosomal proteinases.. Biol. Chem.

[35] Rossi A, Deveraux Q, Turk B, Sali A (2004). Comprehensive search for cystein cathepsin in the human genome.. Biol. Chem.

[36] Sardiello M, Palmieri M, di Ronza A, Medina DL, Valenza M (2009). A gene network regulating lysosomal biogenesis and function.. Science.

[37] Mizushima N, Yoshimori T (2007). How to interpret LC3 immunoblotting.. Autophagy.

[38] Al-Younes HM, Brinkmann V, Meyer TF (2004). Interaction of *Chlamydia trachomatis* serovar L2 with the host autophagic pathway.. Infect. Immun.

[39] Komatsu M, Waguri S, Ueno T, Iwata J, Murata S (2005). Impairment of starvation-induced and constitutive autophagy in Atg7-deficient mice.. J. Cell Biol.

[40] Gieffers J, Belland RJ, Whitmire W, Ouellette S, Crane D (2002). Isolation of *Chlamydia pneumoniae* clonal variants by a focus-forming assay.. Infect. Immun.

[41] Pachikara N, Zhang H, Pan Z, Jin S, Fan H (2009). Productive *Chlamydia trachomatis* lymphogranuloma venereum 434 infection in cells with augmented or inactivated autophagic activities.. FEMS Microbiol. Lett.

[42] Pisoni RL, Thoene JG (1991). The transport systems of mammalian lysosomes.. Biochim. Biophys.Acta.

[43] Thamotharan M, Lombardo YB, Bawani SZ, Adibi SA (1997). An active mechanism for completion of the final stage of protein degradation in the liver, lysosomal transport of dipeptides.. J. Biol. Chem.

[44] Doeven MK, van den Bogaart G, Krasnikov V, Poolman B (2008). Probing receptor-translocator interactions in the oligopeptide ABC transporter by fluorescence correlation spectroscopy.. Biophys. J.

[45] Skipp P, Robinson J, O'Connor CD, Clarke IN (2005). Shotgun proteomic analysis of *Chlamydia trachomatis*.. Proteomics.

[46] Miyairi I, Mahdi OS, Ouellette SP, Belland RJ, Byrne GI (2006). Different growth rates of *Chlamydia trachomatis* biovars reflect pathotype.. J. Infect. Dis.

[47] Ouellette SP, Hatch TP, Abdel-Rahman YM, Rose LA, Belland RJ (2006). Global transcriptional upregulation in the absence of increased translation in *Chlamydia* during IFN gamma-mediated host cell tryptophan starvation.. Mol. Microbiol.

[48] Drecktrah D, Knodler LA, Howe D, Steele-Mortimer O (2007). *Salmonella* trafficking is defined by continuous dynamic interactions with the endolysosomal system.. Traffic.

[49] Seshadri R, Paulsen IT, Eisen JA, Read TD, Nelson KE (2003). Complete genome sequence of the Q-fever pathogen *Coxiella burnetii*.. Proc. Natl. Acad. Sci.USA.

[50] Kuijl C, Savage NDL, Marsman M, Tuin AW, Janssen L (2007). Intracellular bacterial growth is controlled by a kinase network around PKB/AKT1.. Nature.

[51] Byrne GI, Ouellette SP, Wang Z, Rao JP, Lu L (2001). *Chlamydia pneumoniae* expresses genes required for DNA replication but not cytokinesis during persistent infection of HEp-2 cells.. Infect Immun.

[52] MacLean KH, Dorsey FC, Cleveland JL, Kastan MB (2008). Targeting lysosomal degradation induces p53-dependent cell death and prevents cancer in mouse models of lymphomagenesis.. J. Clin. Invest.

[53] Dorsey FC, Steeves MA, Prater SM, Schröter T, Cleveland JL (2009). Monitoring the autophagy pathway in cancer.. Methods Enzymol.

