# Supplementary figures and images for: Chlamydia Species-Dependent Differences in the Growth Requirement for Lysosomes
Source: PLoS One. 2011 Mar 8;6(3):e16783. doi: 10.1371/journal.pone.0016783 (PMC3050816; doi:10.1371/journal.pone.0016783)

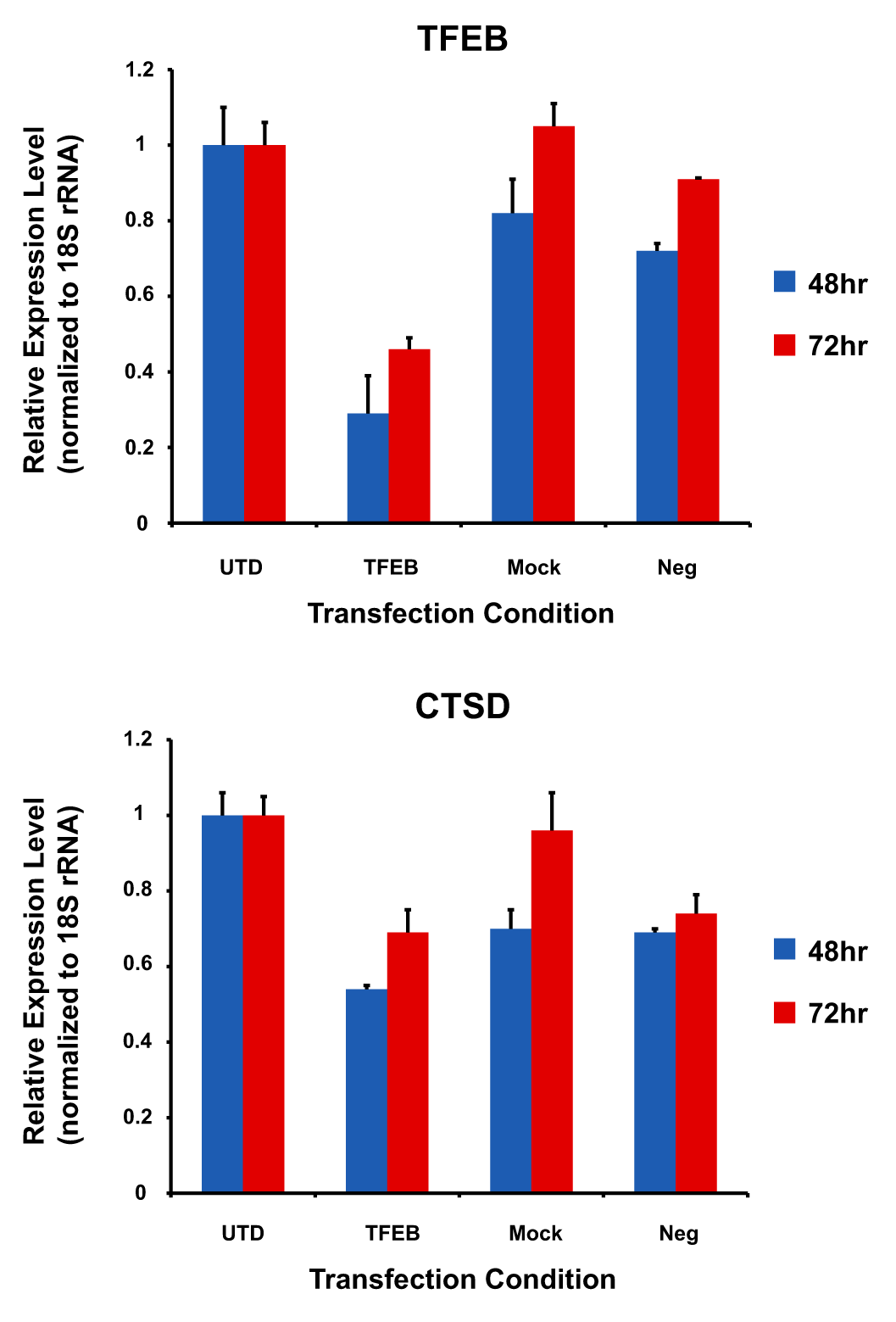

Supplement: Figure S1 — Expression of TFEB and CTSD over time during siRNA knockdown experiments. Cells were transfected as indicated or untransfected (UTD) and total RNA was collected. Expression of the genes was monitored by qPCR and normalized to 18S rRNA levels as described in Materials and Methods. (TIF) [file pone.0016783.s001.tif]
